# Synthesis, Characterization and Cytotoxicity of New Rotundic Acid Derivatives

**DOI:** 10.3390/molecules17021278

**Published:** 2012-01-31

**Authors:** Yu-Fang He, Min-Lun Nan, Jia-Ming Sun, Zhao-Jie Meng, Fa-Gui Yue, Quan-Cheng Zhao, Xiao-Hong Yang, Hui Wang

**Affiliations:** 1 School of Pharmaceutical Sciences, Jilin University, Changchun 130021, China; Email: he_yufang1992@163.com (Y.-F.H.); agui_228@126.com (F.-G.Y.); 2 Jilin Academy of Chinese Medicine Sciences, Changchun 130012, China; Email: nanminlun2000@163.com (M.-L.N.); zhaoquancheng1954@126.com (Q.-C.Z.); 3 Development Center of Traditional Chinese Medicine and Bioengineering, Changchun University of Chinese Medicine, Changchun 130117, China; Email: sun_jiaming2008@163.com; 4 Norman Bethune College of Medicine, Jilin University, Changchun 130021, China; Email: mengzhaojie5555@163.com; 5 China-Japan Union Hospital, Jilin University, Changchun 130033, China

**Keywords:** rotundic acid, amino acid derivative, synthesis, characterization, cytotoxicity

## Abstract

Rotundic acid (**RA**, **1**), a natural compound, exhibits potent tumor cell growth inhibiting properties. To date there are no reports on derivatives of **RA**. Furthermore, the 28-COOH position of **RA** might make it unstable and induced serious gastrointestinal side effects when it was applied *in vivo*. Therefore, in order to explore and make use of this compound, eight new amino acid derivatives of **RA** at the 28-COOH position were synthesized and evaluated for their cytotoxicities *in vitro* on three tumor cell lines including A375, HepG2 and NCI-H446. As a result, a few of these new amino acid derivatives showed stronger cytotoxicity. Compound **5a** was found to have the best inhibition activity on the three tested human tumor cell lines with IC_50_ values of less than 10 μM compared with **RA** treatment. Meanwhile, the cytotoxicity of compound **6b** was significantly higher than that of **RA** on the A375 cell line and almost the same as **RA** on the HepG2 and NCI-H446 cell lines. Hence, compounds **5a** and **6b** may serve as potential lead compounds for the development of new anti-tumor drugs.

## 1. Introduction

**Figure 1:**
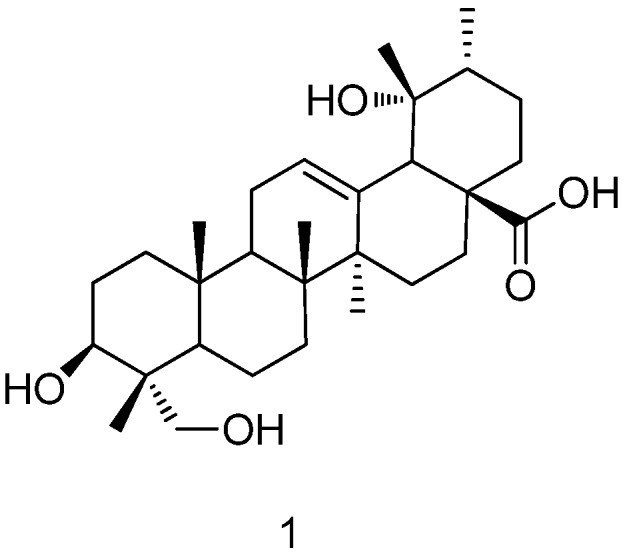
Structure of rotundic acid (**RA**).

Rotundic acid (**RA**, **1**, Figure 1) belongs to the pentacyclic triterpenoid family and is mainly found in *Ilex rotunda*, *Ilex* *purpurea*, *Ilex integra* and other *Aquifoliaceae* plants which are widely distributed in China [1,2,3,4]. **RA** was also isolated from *Mussaenda Pubescens* and *Guettarda platypoda* of the *Rubiaceae* family [5,6]. *Olea europaea* and *Planchonella duclitan*, which are part of the *Oleaceae* and *Sapotaceae* families, respectively, also contain **RA**[7,8]. Although there are sufficient sources for extraction of **RA** in China, as mentioned above, there are still few reports on its bioactivity because of little interest from pharmacological researchers. In our open patent, a considerable amount of **RA** was isolated and purified from *I*. *rotunda* [9]. Moreover, Xu *et al.* demonstrated that **RA**, as one of many isolated compounds, showed anti-cancer activity [10]. Li *et al.* also reported that **RA**showed cytotoxicity, with IC_50_ values of 21.8 μM and 9.5 μM when it was applied on the HT29 and MCF-7 cell lines, respectively [8]. However, they did not continue to pay much attention to this compound. Since **RA** might be a potential native anticancer drug with sufficient sources, our research group has investigated and applied for a series of patents regarding **RA** and its derivatives during the past few years to explore and make use of this compound [11,12,13,14,15]. 

It has been widely reported that compounds with free carboxylic acids might be unstable during metabolic processes and further induce serious gastrointestinal side effect in humans. Although **RA** presented potential anti-tumor activity, **RA** with its free carboxylic acid might have the same problems when administrated *in vivo* [16]. Currently, structure modification is considered to be an effective method to produce lead compounds to enhance the activity and avoid possible side effects. Moreover, the structure of **RA** is comparatively simple, with a few active positions available for modification. These chemical modifications could be controlled easily, which would make it possible to explore new compounds with better anti-tumor activities. In this work, we carried out structure modification at the 28-COOH position of **RA** to improve the bioactivity of **RA** according to the theory of medicinal chemistry and with the experience of structural modification of pentacyclic triterpenoids [17,18,19,20].

Amino acids, as the basis of all metabolic cycles, are the essential compounds responsible for all life. There is a sizeable amount of literature that shows that tumor cells require larger quantities of amino acids than normal cells in the body [21,22]. Hence, in theory the selectivity of a drug for tumor cells may improve when amino acids are introduced into the drug’s molecular structure. Many researchers have given much attention to the investigation of the bioactivities of amino acid drugs. Recently, many anti-tumor drugs have exhibited increased selectivity of tumor cells after undergoing amino acid modification. Their anti-tumor activities have been markedly improved and the toxicity on normal cells was lowered [23,24,25].

To the best of our knowledge, there are few reports on the bioactivity of **RA** and no reports on its derivatives. Therefore, the objective of our present study was to investigate the synthesis, characterization, and cytotoxicity of some new **RA** derivatives produced via introduction of amino acid groups. Their structures were elucidated on the basis of spectroscopic assays such as IR, MS, ^1^H-NMR and ^13^C-NMR. The MTT assay was employed to screen their cytotoxicity on the A375, HepG2 and NCI-H446 human cell lines. 

## 2. Results and Discussion

### 2.1. Preparation of **RA**

The procedure reported by Xu *et al.*, for isolating **RA** from *I. rotunda* [10] was followed. Briefly, the barks of *I. rotunda* were shade-dried, ground, and extracted with refluxing 80% EtOH. The EtOH extract was evaporated ubder vacuum to obtain the total saponins fraction. The air-dried and powdered total saponins were hydrolyzed by 4% NaOH in 30% EtOH and purified by recrystallization to prepare **RA**. The purity of **RA** used was ≥98% (HPLC assay). The extraction yield of **RA** in our study was much higher, up to 100 mg/g, which made it suitable for industry production. 

### 2.2. Structure Modification of **RA**

**Scheme 1:**
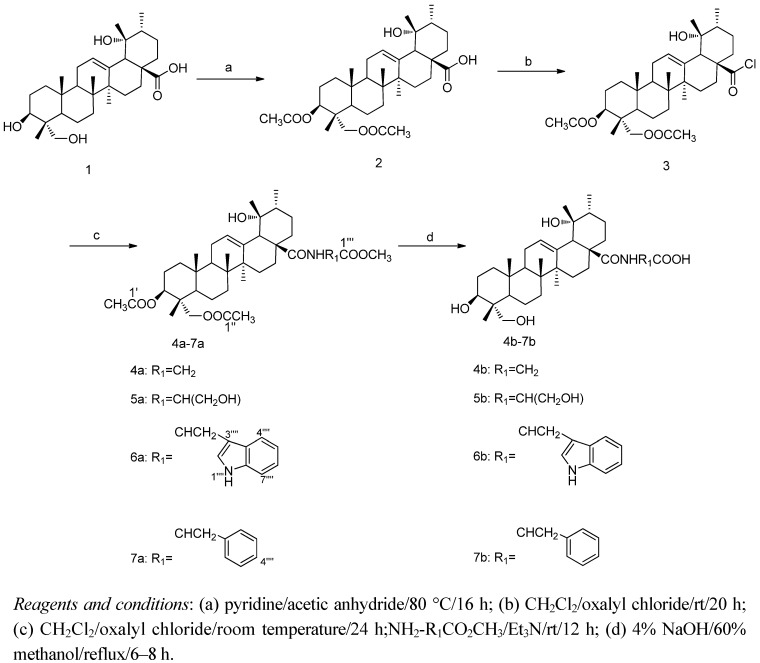
Synthesis of **RA** derivatives.

In the present study, the synthetic routes to the **RA** amino acid derivatives are outlined in Scheme 1. Firstly, **RA** (**1**) was converted to its 3,23-*O*-diacetate **2**, which was then treated with oxalyl chloride to give the 28-acyl chloride **3**. This intermediate was then reacted with the appropriate amino methyl ester hydrochloridea (glycine methyl ester hydrochloride, L-serine methyl ester hydrochloride, L-tryptophan methyl ester hydrochloride, L-phenylalanine methyl ester hydrochloride) in the presence of methylene chloride to give the *N*-[3β,23-diacetoxy-19α-hydroxyurs-12-en-28-oyl]-amino acid methyl esters **4a**–**7a**. Hydrolysis of compounds **4a**–**7a** gave the corresponding *N*-[3β,19α,23-trihydroxyurs-12-en-28-oyl]-amino acids **4b**–**7b**. The structures of these synthesized compounds were confirmed by infrared (IR), mass spectra (MS), ^1^H-NMR and ^13^C-NMR [26,27,28,29]. All eight compounds obtained here were synthesized in high yields with purities of 98% or better and are reported for the first time.

It has been broadly reported that amino acid modification could enhance the anticancer activities of original compounds. Zhuo *et al.* demonstrated that amino acid derivatives of 5-fluorouracil had higher anti-tumor activity with lower toxicity; some of them reached 90% inhibition rate in Ehrlich carcinoma or sarcoma in mice [30,31,32,33,34]. Sun *et al.* designed and synthesized a series of amino acid conjugates of 3-oxooleanolic acid, and determined their anti-tumor activities *in vitro*. Preliminary anti-tumor bioassayd showed that conjugates with higher water solubility retained anti-tumor activity [35]. In the present study, we modified the 28-COOH position of **RA** whereby eight new compounds were obtained. Since the amino acid modification might enhance the antitumor activities of original compound as reported, the pharmacological activity of **RA** and its eight derivatives were tested in the following study.

### 2.3. Biological Activity

**Figure 2:**
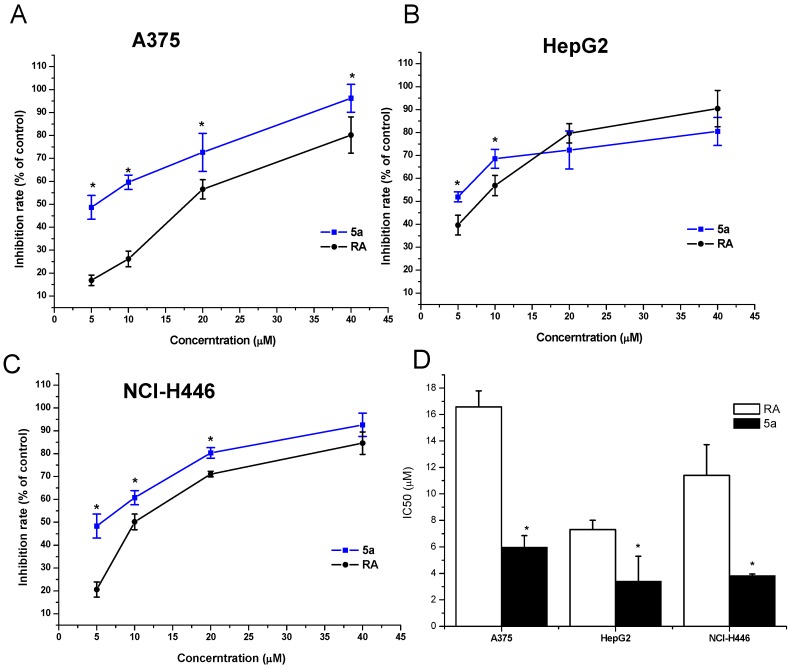
Inhibitory effect of **5a** on the human cancer cell proliferation. (**A**) A375; (**B**) HepG2; (**C**) NCI-H446; (**D**) IC_50_ of **RA** and compound **5a**, * *p* < 0.05 *vs.* **RA**.

In the present study, three types of human cancer cell lines including A375 (human malignant melanoma cells), HepG2 (human hepatoma cells) and NCI-H446 (human small cell lung cancer) were used to observe the cytotoxicity of **RA** (as a positive control) and its derivatives **4a**–**7a**, **4b**–**7b**. Antiproliferative effects were determined with the MTT assay [36]. Each experiment was repeated at least three times. The results are shown in Table 1 and Figure 2.

**Table 1:** The IC_50_ values of RA and its derivatives **4a–7a**, **4b–7b** on human cancer cell lines (μM).

Compound	R1	IC_50_ ± SD (µM)		
		A375	HepG2	NCI-H446
**RA**	–	16.58 ± 1.22	7.33 ± 0.68	11.40 ± 2.32
**4a**	CH_2_	27.97 ± 2.55	10.73 ± 1.69	14.79 ± 3.10
**5a**	CH(CH_2_OH)	5.99 ± 0.88 *	3.41 ± 1.89 *	3.84 ± 0.12 *
**6a**	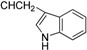	20.60 ± 0.67	44.39 ± 2.87	41.78 ± 2.36
**7a**	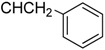	23.12 ± 1.23	85.70 ± 3.55	20.84 ± 3.69
**4b**	CH_2_	>100 ^a^	46.67 ± 3.98	15.24 ± 1.58
**5b**	CH(CH_2_OH)	>100 ^a^	22.28 ± 2.25	82.79 ± 2.98
**6b**	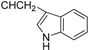	8.03 ± 0.87 *	6.11 ± 1.00	11.32 ± 1.56
**7b**	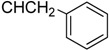	34.59 ± 1.96	14.19 ± 0.98	11.99 ± 1.48

Notes: Data are represented in mean ± SD; *n* = 3. ^a^ IC_50_ values more than 100 µM are indicated as >100,* *p* < 0.05 *vs*. **RA**.

As shown in Table 1, **RA** showed significant IC_50_ values of 16.58, 7.33, 11.40 μM on A375, HepG2, and NCI-H446, respectively, which is consistent with the previous results by Li *et al.* [8] and Xu *et al.* [10], who investigated the cytotoxicity of **RA** on HeLa, MDA-MB-435, CNE1, HT29b, MCF-7c *etc*. Based on the cytotoxicity of **RA**, we tested the cytotoxicity of the compounds **4a**–**7a**, **4b**–**7b**. The result demonstrated that compounds **4a** and **5a**presented more potent anti-tumor activity on the A375, HepG2 and NCI-H446 cell lines compared to compounds **4b** and **5b**. Interestingly, when comparing compounds **6a** and **7a** to compounds **6b** and **7b**, the results were just the opposite. The compounds **6b** and **7b** presented more potent anti-tumor activity on the A375, HepG2 and NCI-H446 cell lines compared to compounds **6a** and **7a** (Table 1). The results might be explained by a steric hindrance arising from the conjugation of an amino group at C-28.When the group is an amino acid methyl ester, the activity of a small steric group is more potent than that of a large one, but when the group is an amino acid, a large steric hindrance is more effective than a small one. In addition, we can see from Table 1 that the cytotoxicity of **6b** was similar to that of the **RA** treatment on the HepG2 and NCI-H446 cell lines, and significantly higher than the **RA** treatment on the A375 cell line (8.03 μM *vs*. 16.58 μM). Also, the IC_50_ of **6b** on HepG2 was also less than 10 μM (6.11 μM), but it was not significantlt different when compared to the IC_50_ of **RA**. These results demonstrate that compound **6b** might be a potential anticancer drug and this will require further investigation. Furthermore, as shown in Figure 2, the inhibitory rates of compound **5a** on the three cell lines were significantly increased at low concentration (5.0 and 10.0 μM) compared to **RA** treatment in a dose dependent manner. The IC_50_ of compound **5a** was significantly less than **RA** treatment group (<10 μM), which indicated that compound **5a** could be a candidate for the development of new anticancer drugs. It was well known that hydroxyl groups can increase water solubility which might enhance the activity of the compound (the IC_50_ values of compound **5a**on the three cell lines were 5.99, 3.41, and 3.84 μM, respectively). However, further investigation of compound **5a** still needs to be conducted and could include studying its anticancer activity *in vivo*. 

## 3. Experimental

### 3.1. General

Reagent-grade chemicals and solvents were obtained from commercial suppliers. Melting points were determined on a Fisher-Johns apparatus and are uncorrected. IR spectra were recorded on a Perkin-Elmer 983G spectrometer. NMR spectra were measured in C_5_D_5_N on a Bruker AM-400 spectrometer, using TMS as an internal standard. NMR experiments included the HMQC and HMBC pulse sequences. Coupling constants (*J* values) are given in Hz, and a MS Agilent 1100 Series LC/MSD ion-trap mass spectrometer was used to record the ESI-MS and HR-ESI-MS. All solvents were freshly distilled and dried prior to use, according to the standard procedures. All chemicals were purchased from Sigma Chemicals Ltd. The human hematoma cell line (HepG2), human malignant melanoma cell line (A375) and human small cell lung cancer cell line (NCI-H446) were purchased from Jilin Provincial Tumor Hospital.

### 3.2. Extraction and Isolation of **RA** (**1**)

The barks (1.0 kg) of *I. rotunda* were shade-dried, ground, and extracted with refluxing 80% EtOH successively (8 L, 3 h, two times). The EtOH extract was evaporated *in vacuo* to yield total saponins (100 g). The air-dried and powdered total saponins (100.0 g) were refluxed with 4% NaOH in 30% EtOH (5.0 L) at 100 °C for 4 h. The mixture was then cooled to room temperature and extracted with EtOAc (1.0 L × 3). The combined organic layers were concentrated under reduced pressure to give the residue (47.1 g), which was recrystallized by MeOH-H_2_O to yield pure **RA** (32.3 g). mp 272.0–273.5 °C; IR (KBr) cm^−1^: 3570, 3417, 2933, 2878, 1689, 1460, 1388, 1046 and 933; ^1^H-NMR δ:5.50 (1H, m, H-12), 4.84 (1H, m, H-3), 4.07 (1H, m, H-23a), 3.60 (1H, d, *J* = 10.3 Hz, H-23b), 2.97 (1H, br s, H-18), 1.57 (3H, s, CH_3_-25), 1.32 (3H, s, CH_3_-27), 1.02 (3H, s, CH_3_-29), 1.00 (3H, d, *J* = 6.64 Hz, CH_3_-30), 0.95 (3H, s, CH_3_-26), 0.88 (3H, s, CH_3_-24); ^13^C-NMR δ: 180.8 (C-28), 140.1 (C-13), 128.2 (C-12), 73.7 (C-3), 72.8 (C-19), 68.2 (C-23), 54.8 (C-18), 48.8 (C-5), 48.4 (C-9), 47.9 (C-17), 43.0 (C-20), 42.5 (C-14), 42.3 (C-8), 40.5 (C-1), 39.0 (C-4), 38.6 (C-22), 37.3 (C-10), 33.4 (C-7), 29.5 (C-15), 27.8 (C-21), 27.2 (C-29), 27.0 (C-2), 26.5 (C-16), 24.8 (C-27), 24.2 (C-11), 18.9 (C-6), 17.4 (C-25), 16.9 (C-26), 16.1 (C-30), 13.2 (C-24). ESI-MS *m/z*: 489.4 [M+H]^+^.

### 3.3. General Procedure for the Preparation of 19α-Hydroxy-3β, 23-diacetoxyurs-12-en-28-oic acid (**2**)

**RA** (1.02 mmol) was dissolved in pyridine (20 mL), then acetic anhydride (10 mL) was added to mixture which was stirred at 80 °C for 16 h. The solvent was removed under reduced pressure using a rotary evaporator. The residue was washed with water, and evaporated to dryness. The residue was purified by column chromatography on silica gel to give compound **2** as colorless needles. Yield, 69.9%; mp 156.5–158.5 °C; IR (KBr) cm^−1^: 3597, 3443, 2955, 2873, 1727, 1704, 1472, 1370, 1036 and 924; ^1^H-NMR δ: 5.47 (1H, m, H-12), 4.97 (1H, m, H-3), 4.89 (1H, m, H-23a), 3.89 (1H, brs, H-23b), 2.93 (1H, br s, H-18), 1.91 (3H, s, CH_3_-1'), 1.86 (3H, s, CH_3_-1''),1.63 (3H, s, CH_3_-25), 1.32 (3H, s, CH_3_-27), 0.97 (3H, s, CH_3_-29), 1.00 (3H, d, *J* = 6.64 Hz, CH_3_-30), 0.76 (3H, s, CH_3_-26), 0.71 (3H, s, CH_3_-24); ^13^C-NMR δ: 180.8 (C-28), 140.1 (C-13), 127.9 (C-12), 74.8 (C-3), 72.8 (C-19), 66.8 (C-23), 54.7 (C-18), 48.5 (C-5), 48.4 (C-9), 47.9 (C-17), 42.5 (C-20), 42.2 (C-14), 41.0 (C-8), 40.4 (C-1), 38.6 (C-4), 38.0 (C-22), 37.0 (C-10), 33.2 (C-7), 29.4 (C-15), 27.2 (C-21), 27.0 (C-29), 26.5 (C-2), 24.6 (C-16), 24.0 (C-27), 23.5 (C-11), 18.5 (C-6), 17.3 (C-25), 16.9 (C-26), 16.0 (C-30), 13.3 (C-24), 170.7 (C-1'), 21.2 (C-2'), 170.6 (C-1''), 20.8 (C-2''). ESI-MS *m/z*: 573.1 [M+H]^+^. HR-ESI-MS found: 573.3804. calcd: 573.3791 for C_34_H_53_O_7_ ([M+H]^+^).

### 3.4. General Procedure for the Preparation of N-[3β,23-diacetoxy-19α-hydroxy urs-12-en-28-oyl]-amino acid methyl esters **4a–7a**

To a solution of compound **2** (2.33 mmol) in CH_2_Cl_2_ (25 mL) added oxalyl chloride (2 mL) and the mixture was stirred at an ice-water bath for 1 h, then further stirred at room temperature for 24 h. The mixture was concentrated to dryness under reduced pressure (30 °C). CH_2_Cl_2_ was added to the residue three times (each time 50 mL), then the concentrated to dryness to yield crude 3,23-*O*-diacetylursolyl chloride **3**. Next in an ice-water bath, glycine methyl ester hydrochloride (12 mmol, which was dissolved in 60 mL CH_2_Cl_2_ and 6 mL triethylamine) was added to a CH_2_Cl_2_ solution (90 mL) of **3** (2.33 mmol) The reaction mixture was stirred in the ice-water bath for 0.5 h, and then stirred at room temperature for 24 h, and then washed in turn with 2.5% hydrochloric acid, water, and saturated sodium chloride solution (each liquid three times, each time 50 mL). Then the reaction mixture was treated with anhydrous sodium sulfate, filtered, concentrated, and then dried to yield a light yellow solid that was recrystallized from 95% ethanol (400 mL) to yield a white solid. The solid was purified on a silica gel column with petroleum ether and ethyl acetate as eluents to yield white needles. 

#### 3.4.1. *Methyl N-[3β,23-diacetoxy-19α-hydroxy-urs-12-en-28-oyl]-2-amino acetate* (**4a**,C_37_H_57_NO_8_, R_1_ = CH_2_)

Yield 56.9%, mp 198 ~ 200 °C; IR (KBr) cm^−1^: 3411, 2972, 2925, 2882, 1741, 1727, 1651, 1520, 1474, 1444, 1386, 1370, 1250, 1049, 1028 and 1004;^1^H-NMR δ: 8.07 (1H, brs, -NH), 5.44 (1H, m, H-12), 4.93 (1H, m, H-3), 4.25 (1H, m, H-2''' a), 4.10 (1H, m, H-2''' b), 3.93 (1H, d, *J* = 11.6 Hz, H-23a), 3.87 (1H, d, *J* = 11.6 Hz, H-23b), 3.51 (3H, s, 1'''-OCH_3_), 2.82 (1H, br s, H-18), 1.91 (3H, s, CH_3_-2'), 1.86 (3H, s, CH_3_-2''), 1.60 (3H, s, CH_3_-25), 1.27 (3H, s, CH_3_-27), 0.87 (3H, s, CH_3_-29), 0.95 (3H, d, *J* = 6.64 Hz, CH_3_-30), 0.82 (3H, s, CH_3_-26), 0.74 (3H, s, CH_3_-24); ^13^C-NMR δ: 178.7 (C-28), 140.0 (C-13), 128.1 (C-12), 74.8 (C-3), 73.1 (C-19), 65.7 (C-23), 54.4 (C-18), 48.5 (C-5), 48.1 (C-9), 47.9 (C-17), 42.3 (C-20), 42.1 (C-14), 41.0 (C-8), 40.4 (C-1), 38.9 (C-4), 38.0 (C-22), 37.1 (C-10), 33.2 (C-7), 28.9 (C-15), 27.2 (C-21), 27.1 (C-29), 26.2 (C-2), 24.6 (C-16), 24.0 (C-27), 23.5 (C-11), 18.5 (C-6), 17.1 (C-25), 16.9 (C-26), 16.0 (C-30), 13.3 (C-24), 170.7 (C-1'), 21.2 (C-2'), 170.5 (C-1''), 20.8 (C-2''), 171.6 (C-1'''), 51.8 (1'''-OCH_3_), 42.0 (C-2'''). ESI-MS *m/z*: 644.4 [M+H]^+^. HR-ESI-MS found: 644.4122. calcd: 644.4157 for C_37_H_58_NO_8_ ([M+H]^+^).

#### 3.4.2. *Methyl N-[3β,23-diacetoxy-19α-hydroxy -urs-12-en-28-oyl]-2-amino-3-hydroxypropionate* (**5a**, C_38_H_59_NO_9_, R_1_ = CH(CH_2_OH)

Compound **2** was reacted with L-serine methyl ester using general procedure to give compound **5a**. Eluted with petroleum ether/ethyl acetate (V/V) = 5:5. Colorless white needles, yield 64.2%, mp 143 ~ 145 °C; IR (KBr) cm^−1^: 3443, 2935, 2879, 1744, 1653, 1509, 1470, 1371, 1248, 1035 and 935; ^1^H-NMR δ: 7.42 (1H, brs, -NH), 5.50 (1H, m, H-12), 5.03 (1H, m, H-2'''), 4.93 (1H, m, H-3), 4.25 (2H, m, H-3'''), 3.90 (1H, br s, H-23), 3.59 (3H, s, 1'''-OCH_3_), 2.75 (1H, br s, H-18), 1.92 (3H, s, CH_3_-2'), 1.85 (3H, s, CH_3_-2''), 1.59 (3H, s, CH_3_-25), 1.25 (3H, s, CH_3_-27), 0.93 (3H, d, *J* = 6.64 Hz, CH_3_-30), 0.85 (3H, s, CH_3_-29), 0.80 (3H, s, CH_3_-26), 0.74 (3H, s, CH_3_-24); ^13^C-NMR δ: 178.2 (C-28), 139.7 (C-13), 128.5 (C-12), 74.7 (C-3), 73.0 (C-19), 65.7 (C-23), 54.6 (C-18), 48.5 (C-5), 48.1 (C-9), 47.9 (C-17), 42.2 (C-20), 42.1 (C-14), 41.0 (C-8), 40.5 (C-1), 38.9 (C-4), 38.0 (C-22), 37.1 (C-10), 33.2 (C-7), 28.9 (C-15), 27.1 (C-21), 27.0 (C-29), 26.2 (C-2), 24.5 (C-16), 24.1(C-27), 23.5 (C-11), 18.5 (C-6), 17.1 (C-25), 16.8 (C-26), 16.0 (C-30), 13.3 (C-24), 170.7 (C-1'), 21.2 (C-2'), 170.5 (C-1''), 20.8 (C-2''), 172.6 (C-1'''), 62.9 (C-3'''), 56.2 (C-2'''), 52.2 (1'''-OCH_3_). ESI-MS *m/z*: 674.5 [M+H]^+^. HR-ESI-MS found: 674.4222. calcd: 674.4263 for C_38_H_60_NO_9_ ([M+H]^+^).

#### 3.4.3. *Methyl N-[3β,23-diacetoxy-19α-hydroxy-urs-12-en-28-oyl]-2-amino-3-(1H-indol-3-yl)propionate* (**6a**, C_46_H_64_N_2_O_8_, R_1_ =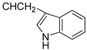 )

Compound **2** was reacted using the general procedure with L-tryptophan methyl ester to give compound **6a**. Eluted with petroleum ether/ethyl acetate (V/V) = 5:2. White needles, yield 54.5%, mp 243 ~ 245 °C; IR (KBr) cm^−1^: 3410, 3372, 2966, 2952, 2878, 1730, 1651, 1519, 1460, 1444, 1368, 1250, 1019 and 742; ^1^H-NMR δ: 11.9 (1H, s, indole-NH), 7.98 (1H, brs, amide-NH), 7.48 (1H, brs, H-4''''), 7.46 (1H, s, H-2''''), 7.35 (1H, m, H-7''''), 7.16 (2H, m, H-5'''' and H-6''''), 5.33 (1H, m, H-12), 5.11 (1H, m, H-2'''), 4.91 (1H, m, H-3), 3.90 (2H, m, H-23), 3.64 (3H, s, 1'''-OCH_3_), 3.35 (2H, m, H-3'''), 2.77 (1H, br s, H-18), 1.96 (3H, s, CH_3_-2'), 1.85 (3H, s, CH_3_-2''), 1.50 (3H, s, CH_3_-25), 1.20 (3H, s, CH_3_-27), 0.91 (3H, d, *J* = 6.64 Hz, CH_3_-30), 0.75 (3H, s, CH_3_-29), 0.70 (3H, s, CH_3_-26), 0. 29 (3H, s, CH_3_-24); ^13^C-NMR δ: 178.2 (C-28), 139.7 (C-13), 137.9 (C-9''''), 128.5 (C-8''''), 128.1 (C-12), 124.5 (C-2''''), 122.3 (C-6''''), 119.6 (C-5''''), 119.1 (C-7''''), 112.4 (C-4''''), 110.9 (C-3''''), 74.8 (C-3), 73.0 (C-19), 65.8 (C-23), 54.1 (C-18), 48.4 (C-5), 47.9 (C-9), 47.8 (C-17), 42.3 (C-20), 41.9 (C-14), 41.0 (C-8), 40.1 (C-1), 38.6 (C-4), 38.0 (C-22), 37.0 (C-10), 32.8 (C-7), 28.6 (C-15), 27.2 (C-21), 27.1 (C-29), 26.2 (C-2), 24.5 (C-16), 23.9 (C-27), 23.5 (C-11), 18.6 (C-6), 16.8 (C-25), 16.2 (C-26), 15.9 (C-30), 13.4 (C-24), 170.7 (C-1'), 21.2 (C-2'), 170.6 (C-1''), 20.8 (C-2''), 174.0 (C-1'''), 28.2 (C-3'''), 54.7 (C-2'''), 52.1 (1'''-OCH_3_). ESI-MS *m/z*: 773.5 [M+H]^+^. HR-ESI-MS found: 773.4715. calcd: 773.4735 for C_46_H_65_N_2_O_8_ ([M+H]^+^).

#### 3.4.4. *Methyl N-[3β,23-diacetoxy-19α-hydroxyurs-12-en-28-oyl]-2-amino-3-phenyl propionate* (**7a**, C_44_H_63_NO_8_, R_1_ =
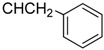
)

Compound **2** was reacted using the general procedure with L-phenylalanine methyl ester to afford compound **7a**. Eluted with petroleum ether/ethyl acetate (V/V) =5:2. White needles, yield 62.0%, mp 145 ~ 147 °C; IR (KBr) cm^−1^: 3603, 3455, 3383, 2940, 2866, 1753, 1728, 1714, 1653, 1518, 1471, 1444, 1379, 1252, 1037, 744 and 696; ^1^H-NMR δ: 7.54 (1H, brs, -NH), 7.28 (4H, m, H-2'''', 3'''', 5'''', and 6''''), 7.20 (1H, m, H-4''''), 5.39 (1H, m, H-12), 5.00 (1H, m, H-2'''), 4.92 (1H, m, H-3), 3.98 (2H, m, H-23), 3.62 (3H, s, 1'''-OCH_3_), 3.15 (2H, m, H-3'''), 2.75 (1H, br s, H-18), 1.92 (3H, s, CH_3_-2'), 1.87 (3H, s, CH_3_-2''), 1.55 (3H, s, CH_3_-25), 1.22 (3H, s, CH_3_-27), 0.93 (3H, d, *J* = 6.64 Hz, CH_3_-30), 0.79 (3H, s, CH_3_-29), 0.76 (3H, s, CH_3_-26), 0. 54 (3H, s, CH_3_-24); ^13^C-NMR δ: 178.4 (C-28), 139.7 (C-13), 138.2 (C-1''''), 129.9 (C-2'''' and 6''''), 129.1 (C-3'''' and 5''''), 128.2 (C-12), 127.3 (C-4''''), 74.8 (C-3), 73.0 (C-19), 65.8 (C-23), 54.2 (C-18), 48.5 (C-5), 48.0 (C-9), 47.9 (C-17), 42.3 (C-20), 42.0 (C-14), 41.0 (C-8), 40.3 (C-1), 38.5 (C-4), 38.0 (C-22), 37.0 (C-10), 32.9 (C-7), 28.8 (C-15), 27.2 (C-21), 27.1 (C-29), 26.1 (C-2), 24.5 (C-16), 24.0 (C-27), 23.5 (C-11), 18.4 (C-6), 16.9 (C-25), 16.8 (C-26), 16.0 (C-30), 13.4 (C-24), 170.7 (C-1'), 21.2 (C-2'), 170.6 (C-1''), 20.8 (C-2''), 173.5 (C-1'''), 28.2 (C-3'''), 55.3 (C-2'''), 52.1 (1'''-OCH_3_). ESI-MS *m/z*: 734.5 [M+H]^+^. HR-ESI-MS found: 734.4888. calcd: 734.4632 for C_44_H_64_NO_8_ ([M+H]^+^).

### 3.5. General Procedure for the Preparation of N-[3β,19α,23-trihydroxy-urs-12-en-28-oyl] amino acids **4b–7b**

A solution of **4a** (or **5a**–**7a**) was stirred and refluxed for 6–8 h with aqueous NaOH (4 %) in 60% CH_3_OH, cooled, water (50 mL) was added, and then treated with 2 N HCl to pH 5, filtered, the solid was washed with water, and dried to give a white powder. 

#### 3.5.1. *N-[3β,19α,23-trihydroxyurs-12-en-28-oyl]-2-amino acetic acid* (**4b**, C_32_H_51_NO_6_, R_1_ = CH_2_)

Yield 83.3%, mp 224 ~ 226 °C; IR (KBr) cm^−1^: 3451, 3369, 2932, 2876, 1634, 1611, 1525, 1460, 1367, 1046 and 932; ^1^H-NMR δ: 7.74 (1H, brs, -NH), 5.53 (1H, brs, H-12), 5.01 (1H, m, H-3), 4.43 (1H, m, H-1''' a), 4.28 (1H, m, H-1''' b), 4.07 (2H, m, H-23), 3.77 (2H, m, H-2'''), 2.80 (1H, br s, H-18), 1.55 (3H, s, CH_3_-25), 1.28 (3H, s, CH_3_-27), 0.97 (3H, s, CH_3_-29), 0.96 (3H, d, *J* = 6.64 Hz, CH_3_-30), 0.97 (3H, s, CH_3_-26), 0.91 (3H, s, CH_3_-24); ^13^C-NMR δ: 178.6 (C-28), 140.1 (C-13), 128.6 (C-12), 73.7 (C-3), 73.1 (C-19), 68.2 (C-23), 54.8 (C-18), 48.8 (C-5), 48.1 (C-9), 48.0 (C-17), 43.0 (C-20), 42.6 (C-14), 42.4 (C-8), 40.6 (C-1), 39.0 (C-4), 39.0 (C-22), 37.3 (C-10), 33.3 (C-7), 29.0 (C-15), 27.8 (C-21), 27.3 (C-29), 27.2 (C-2), 26.4 (C-16), 24.8 (C-27), 24.2 (C-11), 18.9 (C-6), 17.1 (C-25), 16.9 (C-26), 16.2 (C-30), 13.2 (C-24), 173.5 (C-1'''), 42.3 (C-2'''). ESI-MS *m/z*: 546.3 [M+H]^+^. HR-ESI-MS found: 546.3711. calcd: 546.3795 for C_32_H_52_NO_6_ ([M+H]^+^).

#### 3.5.2. *N-[3β,19α,23-trihydroxy urs-12-en-28-oyl]-2-amino-3-hydroxypropionic acid* (**5b**, C_33_H_53_NO_7_, R_1_ = CH(CH_2_OH)

White powder, yield 88.1%, mp 248 ~ 249 °C; IR (KBr) cm^−1^: 3412, 2936, 2876, 1726, 1631, 1515, 1466, 1388, 1350, 1037 and 938; ^1^H-NMR δ: 7.43 (1H, brs, -NH), 5.60 (1H, brs, H-12), 5.15 (1H, m, H-2'''), 5.11 (1H, m, H-3), 4.47 (2H, m, H-3'''), 4.08 (2H, m, H-23), 2.76 (1H, br s, H-18), 1.54 (3H, s, CH_3_-25), 1.25 (3H, s, CH_3_-27), 0.97 (3H, s, CH_3_-29), 0.93 (3H, d, *J* = 6.64 Hz, CH_3_-30), 0.92 (3H, s, CH_3_-26), 0.88 (3H, s, CH_3_-24); ^13^C-NMR δ: 178.3 (C-28), 139.7 (C-13), 129.0 (C-12), 73.7 (C-3), 73.0 (C-19), 68.2 (C-23), 54.9 (C-18), 48.8 (C-5), 48.3 (C-9), 47.9 (C-17), 43.0 (C-20), 42.3 (C-14), 42.2 (C-8), 40.6 (C-1), 39.2 (C-4), 39.0 (C-22), 37.3 (C-10), 33.4 (C-7), 29.1 (C-15), 27.8 (C-21), 27.2 (C-29), 27.2 (C-2), 26.4 (C-16), 24.7 (C-27), 24.3 (C-11), 18.9 (C-6), 17.1 (C-25), 16.8 (C-26), 16.1 (C-30), 13.2 (C-24), 174.5 (C-1'''), 63.5 (C-3'''), 56.6 (C-2'''). ESI-MS *m/z*: 576.4 [M+H]^+^. HR-ESI-MS found: 576.3911. calcd: 576.3900 for C_33_H_54_NO_7_ ([M+H]^+^).

#### 3.5.3. *N-[3β,19α,23-trihydroxyurs-12-en-28-oyl]-2-amino-3-(1H-indol-3-yl)propionic acid* (**6b**, C_41_H_58_N_2_O_6_, R_1_ =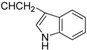)

White powder, yield 88.2%, mp 257 ~ 258 °C; IR (KBr) cm^−1^: 3463, 3411, 2930, 2879, 2862, 1728, 1658, 1499, 1457, 1444, 1387, 1357, 1242, 1042 and 743; ^1^H-NMR δ: 11.9 (1H, s, indole-NH), 7.98 (1H, brs, amide-NH), 7.48 (1H, brs, H-4''''), 7.46 (1H, s, H-2''''), 7.35 (1H, m, H-7''''), 7.16 (2H, m, H-5'''' and H-6''''), 5.33 (1H, m, H-12), 5.11 (1H, m, H-2'''), 4.91 (1H, m, H-3), 3.90 (2H, m, H-23), 3.64 (3H, s, 1'''-OCH_3_), 3.35 (2H, m, H-3'''), 2.77 (1H, br s, H-18), 1.96 (3H, s, CH_3_-2'), 1.85 (3H, s, CH_3_-2''), 1.50 (3H, s, CH_3_-25), 1.20 (3H, s, CH_3_-27), 0.91 (3H, d, *J* = 6.64 Hz, CH_3_-30), 0.75 (3H, s, CH_3_-29), 0.70 (3H, s, CH_3_-26), 0.29 (3H, s, CH_3_-24); ^13^C-NMR δ: 178.2 (C-28), 139.7 (C-13), 137.8 (C-9''''), 129.0 (C-8''''), 128.7 (C-12), 1124.6 (C-2''''), 121.9 (C-6''''), 119.6 (C-5''''), 119.4 (C-7''''), 112.3 (C-4''''), 111.6 (C-3''''), 73.9 (C-3), 73.0 (C-19), 68.3 (C-23), 54.6 (C-18), 48.8 (C-5), 48.0 (C-9), 47.9 (C-17), 43.0 (C-20), 42.3 (C-14), 42.1 (C-8), 40.4 (C-1), 39.0 (C-4), 38.9 (C-22), 37.3 (C-10), 33.1 (C-7), 28.8 (C-15), 27.8 (C-21), 27.2 (C-29), 27.2 (C-2), 26.4 (C-16), 24.7 (C-27), 24.2 (C-11), 18.9 (C-6), 16.9 (C-25), 16.7 (C-26), 16.1 (C-30), 13.2 (C-24), 175.8 (C-1'''), 28.1 (C-3'''), 55.0 (C-2'''). ESI-MS *m/z*: 675.3 [M+H]^+^. HR-ESI-MS found: 675.4295. calcd: 675.4373 for C_41_H_59_N_2_O_6_ ([M+H]^+^).

#### 3.5.4. *N-[3β,19α,23-trihydroxy urs-12-en-28-oyl]-2-amino-3-phenylpropionic acid* (**7b**, C_39_H_57_NO_6_, R_1_ =
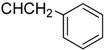
)

White powder, yield 90.1%, mp 219 ~ 221 °C; IR (KBr) cm^−1^: 3195, 3448, 3393, 2978, 2931, 28710, 1753, 1667, 1499, 1456, 1369, 1046 and 705; ^1^H-NMR δ: 7.74 (1H, brs, -NH), 7.42 (2H, m, H-2'''' and 6''''), 7.27 (2H, m, H-3'''' and 5''''), 7.17 (1H, m, H-4''''), 5.46 (1H, m, H-12), 5.12 (1H, m, H-2'''), 4.98 (1H, m, H-3), 4.05 (2H, m, H-23), 3.55 (2H, m, H-3'''), 2.65 (1H, br s, H-18) 1.52 (3H, s, CH_3_-25), 1.20 (3H, s, CH_3_-27), 0.97 (3H, s, CH_3_-29), 0.93 (3H, d, *J* = 6.64 Hz, CH_3_-30), 0.86 (3H, s, CH_3_-26), 0.73 (3H, s, CH_3_-24); ^13^C-NMR δ: 178.3 (C-28), 139.7 (C-13), 138.8 (C-1''''), 130.3 (C-2'''' and 6''''), 128.9 (C-3'''' and 5''''), 128.8 (C-12), 127.2 (C-4''''), 73.8 (C-3), 73.0 (C-19), 68.3 (C-23), 54.7 (C-18), 48.8 (C-5), 48.2 (C-9), 48.0 (C-17), 43.0 (C-20), 42.4 (C-14), 42.2 (C-8), 40.5 (C-1), 39.0 (C-4), 38.8 (C-22), 38.3 (C-10), 37.3 (C-7), 33.1 (C-15), 27.8 (C-21), 27.2 (C-29), 27.2 (C-2), 26.4 (C-16), 24.7 (C-27), 24.2 (C-11), 18.8 (C-6), 16.9 (C-25), 16.9 (C-26), 16.1 (C-30), 13.2 (C-24), 175.2 (C-1'''), 28.9 (C-3'''), 55.4 (C-2'''). ESI-MS *m/z*: 636.3 [M+H]^+^. HR-ESI-MS found: 636.4221. calcd: 636.4259 for C_39_H_58_NO_6_ ([M+H]^+^).

### 3.6. *In Vitro* Anti-tumor Assays

Aliquots (200 µL) of 5 × 10^3^cells per mL of A375, HepG2 and NCI-H446 cells were seeded in 96 well flat-bottomed plates in DMEM medium containing 10% FBS and a penicillin-streptomycin mixture at 37 °C in a humidified atmosphere of 5% CO_2_. The test drugs were dissolved in DMSO. The incubation medium was replaced with each test medium giving a final concentration of 5–40 µmol/L of test compounds and no drug in 2 µL DMSO over 24 h. The ability of the drug to inhibit cellular growth was determined by performing the MTT assay. Each experiment was performed in six wells, and all the experiments involving a control (DMSO only). The drug treatments were performed separately three times. All data are presented as mean ± standard deviations (S.D.). Statistical significance of the differences between groups was assessed by Student's *t*-test.

## 4. Conclusions

To date, though **RA** has been reported to show cytotoxicity, there are no reports on chemical modification of **RA** and the bioactivity of its derivatives. In this work, based on our previous investigation of **RA**, eight novel amino acid derivatives of **RA** were synthesized and their anti-tumor activities were tested *in vitro* for the first time. These eight compounds showed different cytotoxicities on the three tested tumor cell lines. Especially, compound **5a** possesses better activity than **RA**, with 2.76-, 2.14-, 2.96-fold more potent activities than **RA**, respectively. The cytotoxicity of compound **5a**was more sensitive than **RA**, with an IC_50_ value of less than 10 μM on all three cell lines. Compound **6b** showed equivalent activity to **RA** on the HepG2 and NCI-H446 cell lines, and higher cytotoxicity on the A375 cell line. Compounds **5a** and **6b** may thus serve as potential lead compounds for the development of new anticancer drugs. More derivative synthesis and further biological evaluations are currently in progress and will be reported in due course.
